# Effect of Educational Intervention on Knowledge and Level of Adherence among Hemodialysis Patients: A Randomized Controlled Trial

**DOI:** 10.1155/2023/4295613

**Published:** 2023-03-31

**Authors:** Brayal Dsouza, Ravindra Prabhu, B. Unnikrishnan, Sudarshan Ballal, Suneel C. Mundkur, Varalakshmi Chandra Sekaran, Avinash Shetty, Paulo Moreira

**Affiliations:** ^1^Department of Social and Health Innovation, Prasanna School of Public Health, Manipal Academy of Higher Education, Manipal, India; ^2^Department of Nephrology, Kasturba Medical College, Manipal Academy of Higher Education, Manipal, India; ^3^Department of Community Medicine, Kasturba Medical College, Mangalore, Manipal Academy of Higher Education, Manipal, India; ^4^Manipal Hospitals, Manipal Health Enterprises Pvt Ltd, Bengaluru, India; ^5^Department of Paediatrics, Kasturba Medical College, Manipal Academy of Higher Education, Manipal, India; ^6^Department of Health Policy, Prasanna School of Public Health, Manipal Academy of Higher Education, Manipal, India; ^7^Department of Community Medicine, Kasturba Medical College, Manipal Academy of Higher Education, Manipal, India; ^8^International Healthcare Management Research and Development Center (IHM-RDC), Shandong Qianfoshan Provincial Hospital, Shandong Medical University, Jinan, Shandong, China

## Abstract

**Purpose:**

The purpose of the study was to assess the impact of an educational intervention on the level of knowledge and adherence to the treatment regimen among hemodialysis (HD) patients as well as to describe the association between these variables.

**Methods:**

In this randomized controlled trial, 160 HD patients at an HD centre of a 2030-bed tertiary teaching hospital in Southern India were randomly assigned into intervention (*N* = 80, received education and a booklet) and control (*N* = 80, received standard care) groups. Knowledge and adherence were measured preintervention and postintervention using a validated questionnaire for knowledge and the ESRD-AQ (End-Stage Renal Disease Questionnaire) for the level of adherence. The statistical analysis of the data was performed with the help of the Statistical Program SPSS version 19.0. The statistical significance level was set at 0.05.

**Results:**

The increase in knowledge on disease management, fluid adherence, and dietary adherence in the intervention group was significantly higher compared to the control group. There was no significant correlation between knowledge and adherence. Adherence improved for all the domains, i.e., dialysis attendance, episodes of shortening, adherence to medication, fluid restriction, and dietary restriction. Adherence to fluid and dietary restriction was statistically significant. This trail is registered with https://clinicaltrials.gov/ct2/show/CTRI/2018/05/014166.

## 1. Introduction

Patients on dialysis experience assimilating complex treatment regimens, which includes monitoring blood glucose, intradialytic weight gain, bp monitoring, bill burden, physical activity, investigation routine, and adhering to treatment regimens. Patient education is not only a critical mechanism through which patients can have their questions, concerns, and needs regarding kidney disease care addressed but it is also a crucial pathway to ensure that patients can be taught to engage in self-management [1].

Nonadherence among HD patients includes the following, according to the National Kidney Foundation-Kidney Disease Outcomes Quality Initiative (NKF-KDOQI): (a) skipping or reducing the HD session; (b) consuming excessive amounts of potassium- and phosphorus-containing beverages and foods; and (c) failing to take medication as prescribed [2]. Nonadherence to dialysis treatment has been generally reported at rates between 8.5% and 22.1% worldwide [3]. Nonadherence is associated with increased mortality risk (skipping treatment, excessive IDWG, and high phosphate) and with hospitalization risk [4].

Patients' knowledge, either subjective or objective, does not seem to be sufficient. Hence, attention should be paid to supporting patients with more personalized knowledge [5] Some studies have shown that patient knowledge of disease and treatment is associated with an increased level of adherence [6–8]. The success of treatment depends to a large extent on adherence to the strictly recommended therapeutic regimen. To improve adherence, patients' knowledge of disease management should be improved. Some studies have shown that patients' knowledge of disease and treatment is associated with an increased level of adherence [6, 7].

## 2. Need for an Education Program and Rationale for the Current Study

Patients' understanding of hemodialysis and end-stage renal disease (ESRD) is essential for effective self-management and patient outcomes. There is a need for evidence and trials on the effect of therapeutic education among dialysis patients. The available literature on therapeutic interventions of a focused nature has demonstrated positive effects, and evidence on the use of multidisciplinary care lacks certainty and majorly constitutes observational studies and nonrandomized controlled trials.

## 3. Design and Sample

A randomized controlled trial conducted from June 2017 to December 2020 was performed among 160 HD patients at a 2030-bed tertiary teaching hospital using block randomization and allocation concealment, and outcomes assessment was blinded. The criteria for selecting the sample were as follows: (i) HD program two times a week; (ii) above 18 years of age; and (iii) ability to write, read, and understand the local language; patients with cognitive and psychologically different abilities and limited self-care were excluded. The study population was randomly divided into two groups: the intervention group (received an educational intervention and a booklet) and the control group (standard care at the dialysis centre). The educational intervention for each patient was administered for six months with reinforcement and addressing the patient's queries. This was followed up for one year. A postintervention assessment was conducted for knowledge at baseline, half-yearly, and end of 1 year. A postassessment at the end of one year was conducted for the quality of life, adherence level, and health service utilization. The baseline data demographics were collected with a proforma, knowledge of disease management was assessed with a self-administered validated questionnaire and for measurement of the level of adherence, an ESRD-AQ was administered.

## 4. Sample Size Calculation

**Figure 1:**
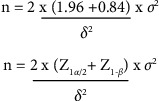
Sample size calculation.

Knowledge was taken as the primary outcome variable for the computation of sample size. A mean difference of 0.5 SD (moderate effect size) is considered clinically important to consider that the intervention is effective. Assuming a power of 80% and a significance level of 5%, the sample size for the comparison of two group means is 64 per group. Adjusting for a 20% dropout rate, the required sample size per group is 80 per group (Figure 1).

(1) $$\begin{array}{c} Sample\;size=60+20\%\;drop\;out=80\;per\;group\text{.} \end{array}$$

## 5. Randomization Procedure

Patients were randomized into two arms, i.e., control and intervention arms, using single block randomization. Block randomization with unequal block sizes of 4 and 6 is used to minimize selection bias, and 1 : 1 allocation will be done for intervention and control groups. For each block in the sequence, the permutation was selected using simple random sampling, and the same was followed for each of the 31 blocks, as shown in the example, and allocation concealment was done. Participants had an equal probability of being assigned to any given group. Finally, 160 patients participated in the study. Each group consisted of 80 patients.

Ethical clearance (441/2015) was obtained from the Institutional Ethics Committee of Kasturba Hospital, MAHE, Manipal, and registered under the Clinical Trial Registry of India's CTRI Registration Number: Trial REF/2017/12/016258. As per the ethical guidelines, a participant information sheet (PIS) and informed consent (IC) for participants were administered.

## 6. Ethical Considerations

Ethical clearance (441/2015) was obtained from the Institutional Ethics Committee of Kasturba Hospital, MAHE, Manipal.

**Figure 2:**
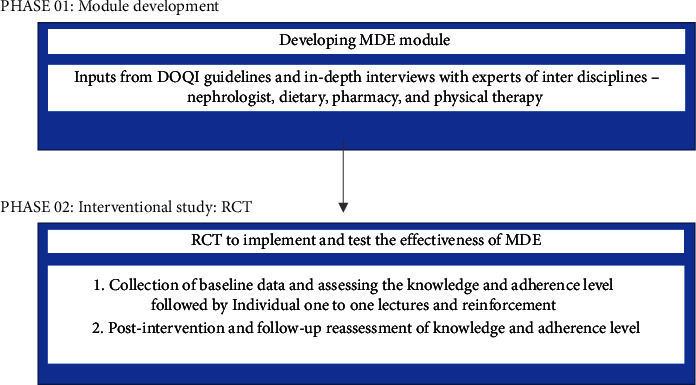
Phases of the study.

**Figure 3:**
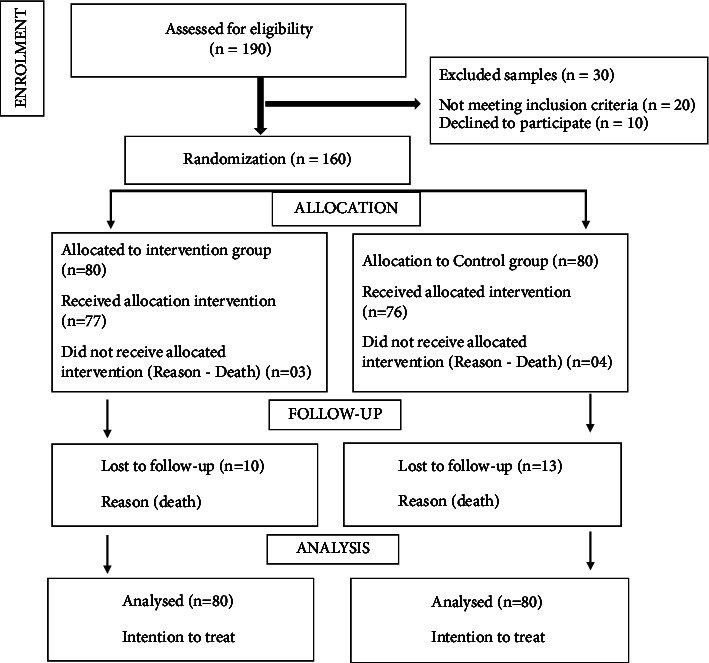
The consort diagram.

*Phase 01* (Figures 2 and 3). Inputs from the KDOQI guidelines and expert' opinion on its adaptation and modification and cultural adaptation to the current population in the study were used to design the educational module. Major adaptations were in nutritional guidelines to the current local population. A judgmental validity was done. Judges who are professionals in the fields of nephrology, nutrition, pharmacy, and physical therapy evaluated this intervention guide. The intervention guide was forwarded to the professionals listed above for feedback. The PI discussed with the specialists the aim and goals of this stage. It was looked for ambiguity, such as ambiguous or badly phrased products, double-barrelled remarks, or jargon. For each item, the percentages of the entire agreement, agreement with small modifications, agreement with large changes, and total disagreement were calculated. Any issue that received 70% or more total disagreement from the experts was removed from the teaching material. All the specialists were alerted to the elements that were in agreement with small and substantial adjustments. Many minor adjustments were fixed with their permission, and some important alterations were altered once a majority of experts agreed to that particular change. The educational materials were written in English and converted to the local language with the back translation before the administration.

*Phase 02*. After the inclusion of the patients into one of the two groups, the following questionnaires were followed:

- Patients were given a questionnaire on sociodemographic and clinical characteristics to characterize patients and identify their background information, and for the assessment of knowledge on disease management, a structured questionnaire.
- The ESRD-AQ explores all dimensions of HD patient adherence

## 7. Statistical Analysis

A mixed ANOVA (repeated measure) was performed to check if there was any significant difference in the average knowledge/adherence across different time points as well as between the intervention group and the control group. A nonparametric approach of mixed ANOVA is performed using the R package “nparLD” to check if there was any significant difference in the average adherence/QoL across different time points as well as between the intervention group and control group as data violated the normality assumptions. As the outcome variable “Knowledge” was not normally distributed, quantile regression was used to determine the factors related to this outcome. As the outcome variable “Adherence” was not normally distributed, quantile regression was used to determine the factors related to this outcome. *p* < 0.05 is considered statistically significant, and analysis is performed using SPSS software.

## 8. Results

The sample characteristics of the study population are described in Table 1. Participants included 80% males in the intervention group and 75.3% males in the control group. A higher proportion of participants in both groups had less than secondary education (52.2% and 43.8%) and were largely unemployed. Vintage of more than a year was comparably higher in both groups. Etiology-wise, hypertension predominated among those in the intervention group (41.3%), while in the control group, diabetes mellitus predominated (56.3%). All participants in the intervention group had comorbidities, while 7 (8.75%) in the control group did not. There was a statistically significant difference between the groups in the etiology of diseases (*p* = 0.001) and the presence of comorbidities (0.002). Regarding the mode of payment for treatment, cash payments were higher in both groups.

There was a significant increment in the knowledge after the intervention (*p* < 0.001) (Table 2). Similarly, also in the control group, there is a significant increment of knowledge score observed potentially due to data contamination and shift changes. It was observed from the between-groups comparison that there was a significant difference in the knowledge between the intervention group and the control group. Adherence to fluid and dietary restriction showed a significant improvement in the intervention group, while in the control group, there was a decrease in adherence (Table 3**).**Adherence for HD attendance, episodes of shortening, adherence to medication, and duration of shortening reported an increasing trend towards improvement; however, this trend is statistically insignificant.

From Table 4, it is observed that the variable “Age” was a significant factor of knowledge. With every unit increase in age, the mean knowledge score reduced, and Table 5 shows individuals with cardiac morbidity and those with no other comorbidities, which were significant factors of adherence. The mean adherence score is more in those who had cardiac comorbidity as compared to other comorbidities.

## 9. Discussion

This study reported that educational intervention can improve knowledge and adherence, by way of improving information, reinforcement, and limiting misconceptions about the disease. Knowledge of disease management and fluid and nutritional adherence improved significantly in the intervention group using an educational/cognitive intervention. Similar other studies using cognitive/educational intervention have improved knowledge on nutritional knowledge and binders, dietary phosphate intake, and weight gain control with a duration of intervention showing effect at minimum 2 months and 6 months with partial positive and positive benefits. The benefits sustained beyond intervention were not reported in these studies [9–12].

Several studies [13, 14] have also shown the positive impact of an educational session on knowledge levels. Ebrahimi et al. [15] also reported a significant increase in the level of their patients' knowledge concerning diet restrictions after an educational intervention. Similar results were found by other researchers [16] in a population of Iranian HD patients.

There was an improving trend in adherence in HD attendance, duration, and episodes of shortening of HD and a statistically significant improvement in fluid and dietary adherence. At baseline, the scores of the first three domains of adherence were reported as high, and this could be attributed to the dialysis centre being the only tertiary-level hospital delivering high-quality care and the patient's perception of the quality of dialysis, while for fluid and dietary compliance, the scores were low due to a lack of knowledge, the myth about food to be avoided and eaten in moderation for their disease condition and the climatic condition making it difficult to adhere to fluid restriction. The adherence outcome assessment was subjective, i.e., as the patient reported, and the educational intervention was followed by goal setting and cognitive behavior therapy (CBT). Similarly, Brantley et al., Chen et al., Reese p et al., Kartvelian et al., Kauric Klein, and Wong et al. improved adherence through educational intervention and subjective measurements such as vascular access cleaning compliance, protein intake compliance, medication adherence by questionnaire, phosphate intake, self-reported BP medication adherence, self-reported dialysis diet, and fluid adherence, respectively [10, 17–21].

Several other studies have used objectives such as biochemical parameter measurements, IDWG, and Kt/V with educational interventions for adherence outcome assessment with negative, positive, and partial positive effects [22–26]. Numerous studies also deployed behavioral interventions/counseling techniques to improve adherence. Cukor et al. adapted CBT and education to improve IDWG, and Cho et al. applied the health contract intervention, which included a formal introduction to the program, mutual goal setting, contracting, and recontracting to support self-care behavior reinforced through praise, encouragement, and support, resulting in a partially positive result. Paysar et al. applied the Bensons relaxation technique with partially positive results. Howren et al. used the self-regulation theory to improve intradialytic weight gain. Hou et al. applied rational emotive therapy and improved IDWG and blood pressure [9, 27–29]. This study reported positive outcomes in adherence through subjective assessment among Indians, while many other similar studies and studies coupled with behavior and affective intervention and outcome assessed subjectively and objectively had diverse and heterogenous outcomes.

Several studies have proven effective, partially effective, or negative results using educational, behavioral, or mixed interventions to improve patient adherence. The current study used an educational intervention and patient-reported compliance to measure the effectiveness and found that an educational intervention can improve knowledge and adherence positively in Indian HD patients similar to previous authors who have demonstrated the importance of health literacy in health systems [30–34]. The increase in knowledge level is not associated with increased adherence.

## 10. Limitation

Patient compliance was purely subjective in nature, and objective measurement of compliance was not performed, e.g., missed dialysis sessions, emergencies, and biochemical parameters. This study was limited to educational/cognitive interventions. Psychologic/affective interventions that appealed to the patient's feelings and emotions or social support and mixed interventions that involved a combination of the abovementioned intervention types were not tested.

The limitation is that the results cannot be generalized as the sample did not come from different regions of India and majorly constituted from coastal Karnataka. Moreover, the impact of factors such as noise, interruption by others, or participants' fatigue may influence the answers of individuals. There was also a limitation of the time available to cover all thematic units, as the participants had only one educational session, which included a variety of thematic sections on CKD. For this reason and to enhance the educational outcome, the booklets were given to each participant separately after the intervention.

## 11. Conclusion

This study was a comprehensive approach and helped to improve the patient's knowledge of disease management and level of adherence. This education module can be used as a nurse-led intervention to improve patients' outcomes.

## Figures and Tables

**Table 1:** Demographic and clinical characteristics of patients.

Variable	Intervention (*n* = 80)	Control (*n* = 80)	*P* value
Gender			
Male	64 (80%)	61 (75.3%)	
Female	16 (20%)	19 (23.75)	0.566
Education			
No education	03 (3.75%)	09 (11.25%)	0.135
Less than higher secondary	42 (52.5%)	35 (43.75%)	
Higher secondary	16 (20%)	19 (23.75%)	
Graduate	16 (20%)	23 (28.75%)	
Post graduate and above	3 (3.75)	4 (5%)	
Employment status			0.164
Employed	23 (28.75)	33 (41.25)	
Unemployed	48 (60%)	43 (53.75)	
Retired	09 (11.25%)	04 (5%)	
Vintage			0.502
>3 months	3 (3.75%)	06 (7.5%)	
3 months to 1 year	20 (25%)	23 (28.75%)	
>1 year	57 (71.25%)	51 (63.75%)	
Mode of payment			0.298
Scheme	11 (13.75%)	8 (10%)	
Cash	42 (52.5%)	42 (52.5%)	
Trust	02 (2.5%)	09 (11.25%)	
Employee State Insurance	16 (20%)	14 (17.5%)	
Ex-servicemen contributory scheme	01 (1.25%)	1 (1.25%)	
Private insurance	08 (10%)	05 (6.25%)	
Employer	0	01 (1.25%)	
Etiology			0.001^*∗*^
Diabetes mellitus	26 (32.5%)	45 (56.25%)	
HTN	33 (41.25%)	8 (10%)	
DM and HTN	03 (3.75%)	6 (7.5%)	
Medication	4 (5%)	11 (13.75%)	
Others	14 (17.5%)	10 (12.5%)	
Comorbidity			0.002^*∗*^
No comorbidity	0	07 (8.75%)	
HTN	48 (60%)	27 (33.75%)	
DM	01 (1.35%)	01 (1.25%)	
DM and HTN	26 (32.5%)	39 (48.75%)	
Cardiac	02 (2.5%)	02 (2.5%)	
Others	03 (3.75%)	04 (5%)	

**Table 2:** Mean scores of knowledge before and after intervention.

Groups	Knowledge: mean (SD)			With group comparison (*p* value)	Between-group comparison (*p* value)
	Pretest *n* = (80)	Posttest_6 m *n* = (80)	Posttest_1 yr *n* = (80)		
Intervention	18.91 (7.02)	20.41 (6.29)	25.00 (4.01)	<0.001^*∗*^	0.044
Control	18.65 (6.27)	19.69 (7.11)	22.14 (7.38)	0.003^*∗*^	

**Table 3:** Mean scores for domain-wise adherence before and after intervention.

Domains	Groups	Range of scores	Adherence: mean SD		With group comparison (*p* value)	Between-group comparison (*p* value)
			Baseline	Follow-up		
HD attendance	Control	100–300	286.25 (33.1)	287.5 (33.2)	0.708	0.187
	Intervention		290 (33.1)	297.5 (15.7)	0.026	

Episode of shortening	Control	0–200	195.0 (15.0)	194.38 (15.8)	0.639	0.320
	Intervention		196.88 (12.1)	198.12 (9.5)	0.349	

Duration of shortening HD	Control	0–100	98.12 (6.6)	98.44 (6.09)	0.704	0.284
	Intervention		97.81 (8.14)	99.37 (3.9)	0.059	

Adherence to medication	Control	0–200	196.25 (13.2)	195 (15.0)	0.566	0.417
	Intervention		196.25 (19.11)	197.5 (17.6)	0.566	

Adherence to fluid restriction	Control	0–200	195.25 (15.0)	193.13 (17.3)	0.657	0.048^*∗*^
	Intervention		180 (28.0)	190 (28.0)	0.019^*∗*^	

Adherence to dietary restriction	Control	0–200	190 (24.3)	163 (69.3)	<0.001^*∗*^	<0.001^*∗*^
	Intervention		164.37 (48.5)	185.62 (31.0)	0.004	

**Table 4:** Regression coefficients based on multiple linear regression-dependent variables: knowledge.

Variable	*P*value	Coefficient	95% confidence interval	
			Lower bound	Upper bound
Intercept	0.000	25.737	14.616	36.858
Age	0.016	−0.122	−0.222	−0.023
Gender				
Female	0.975	0.048	−3.001	3.098
Male		Reference		
Vintage				
<3 months	0.554	−1.711	−7.418	3.996
>1 year	0.322	1.415	−1.401	4.231
3 months to 1 year ref		Reference		
Education				
Graduation	0.763	1.000	−5.538	7.538
Higher secondary	0.331	−3.422	−10.354	3.509
Less than higher secondary	0.324	−3.256	−9.765	3.254
No education	0.327	−3.722	−11.209	3.764
PG and above		Reference		
Employment status				
Employed	0.499	0.959	−1.838	3.756
Retired	0.184	3.244	−1.565	8.054
Unemployed		Reference		
Socioeconomic status				
High	0.429	1.789	−2.674	6.252
Low	0.077	2.489	−0.274	5.251
Middle		Reference		
Comorbidity				
Cardiac	0.805	1.137	−7.944	10.218
Diabetes	0.987	−0.089	−10.799	10.621
HTN	0.813	0.741	−5.442	6.923
HTN and diabetes	0.909	0.367	−5.946	6.679
No comorbidity	0.206	−5.044	−12.893	2.805
Others		Reference		
Etiology				
Diabetes	0.900	0.244	−3.581	4.069
Diabetes and HTN	0.384	−2.752	−8.983	3.480
HTN	0.977	0.059	−3.991	4.110
Medications	0.354	−2.430	−7.598	2.739
Unknown etiology		Reference		

**Table 5:** Regression coefficients based on multiple regression-dependent variables: adherence.

Variable	*P* value	Coefficient	95% confidence interval	
			Lower bound	Upper bound
Intercept	0.000	1130.494	1006.416	1254.573
Age	0.602	−0.272	−1.300	0.756
Gender				
Female	0.775	4.623	−27.338	36.583
Male		Reference		
Vintage				
<3 months	0.222	36.940	−22.554	96.434
>1 year	0.138	22.569	−7.374	52.512
3 months to 1 year ref		Reference		
Education				
Graduation	0.879	−5.546	−77.426	66.334
Higher secondary	0.130	−58.537	−134.600	17.526
Less than higher secondary	0.969	−1.424	−73.810	70.963
No education	0.892	5.698	−76.909	88.305
PG and above		Reference		
Employment status				
Employed	0.803	−3.779	−33.659	26.101
Retired	0.556	15.211	−35.710	66.132
Unemployed		Reference		
Socioeconomic status				
High	0.053	−46.193	−93.003	0.616
Low	0.224	−17.742	−46.442	10.957
Middle		Reference		
Comorbidity				
Cardiac	0.025	108.725	14.131	203.319
Diabetes	0.371	50.364	−60.683	161.411
HTN	0.183	44.274	−21.206	109.754
HTN and diabetes	0.132	50.920	−15.584	117.423
No comorbidity	0.029	94.290	9.991	178.589
Others		Reference		
Etiology				
Diabetes	0.760	6.090	−33.344	45.525
Diabetes and HTN	0.156	−47.313	−112.951	18.326
HTN	0.882	−3.159	−45.034	38.716
Medications	0.819	6.246	−47.496	59.988
Unknown etiology		Reference		
Mode of payment				
Self-pay	0.605	14.207	−40.072	68.487
ECHS	0.503	44.478	−86.391	175.347
Employer	0.485	−62.032	−237.261	113.197
ESI	0.221	−36.232	−94.519	22.054
Private insurance	0.646	−16.179	−85.643	53.285
Scheme	0.752	−10.329	−74.955	54.298
Trust		Reference		

## Data Availability

The data supporting the findings of the current study are available from the corresponding author upon request.

## Abbreviations

CKD: Chronic kidney disease
HD: Hemodialysis
ESRD-AQ: End-Stage Renal Disease Questionnaire
NKF-KDOQI: National Kidney Foundation-Kidney Disease Outcomes Quality Initiative
IDWG: Interdialytic weight gain
RRT: Renal replacement therapy
ANOVA: Analysis of variance
CBT: Cognitive behavior therapy.
